# Substituted anthraquinones represent a potential scaffold for DNA methyltransferase 1-specific inhibitors

**DOI:** 10.1371/journal.pone.0219830

**Published:** 2019-07-15

**Authors:** Rebecca L. Switzer, Jessica Medrano, David A. Reedel, Jill Weiss

**Affiliations:** 1 Department of Chemistry, Bucknell University, Lewisburg, Pennsylvania, United States of America; 2 Program in Cell Biology/Biochemistry, Bucknell University, Lewisburg, Pennsylvania, United States of America; University of Parma, ITALY

## Abstract

In humans, the most common epigenetic DNA modification is methylation of the 5-carbon of cytosines, predominantly in CpG dinucleotides. DNA methylation is an important epigenetic mark associated with gene repression. Disruption of the normal DNA methylation pattern is known to play a role in the initiation and progression of many cancers. DNA methyltransferase 1 (DNMT1), the most abundant DNA methyltransferase in humans, is primarily responsible for maintenance of the DNA methylation pattern and is considered an important cancer drug target. Recently, laccaic acid A (LCA), a highly substituted anthraquinone natural product, was identified as a direct, DNA-competitive inhibitor of DNMT1. Here, we have successfully screened a small library of simplified anthraquinone compounds for DNMT1 inhibition. Using an endonuclease-coupled DNA methylation assay, we identified two anthraquinone compounds, each containing an aromatic substituent, that act as direct DNMT1 inhibitors. These simplified anthraquinone compounds retain the DNA-competitive mechanism of action of LCA and exhibit some selectivity for DNMT1 over DNMT3a. The newly identified compounds are at least 40-fold less potent than LCA, but have significantly less complex structures. Collectively, this data indicates that substituted anthraquinone compounds could serve as a novel scaffold for developing DNMT1-specific inhibitors.

## Introduction

DNA methylation is a critical epigenetic mark associated with gene repression. In mammals, methylation occurs at the 5-position of cytosines, predominately in CpG dinucleotides and methylation patterns vary with cell type [1]. Changes to the normal DNA methylation pattern have been associated with cancer initiation and progression. In cancer cells, hypermethylation of the promoter regions of tumor suppressor genes leads to gene silencing, while global hypomethylation contributes to genetic instability. Unlike DNA mutations associated with tumorigenesis, epigenetic changes are reversible, making the proteins involved in epigenetic gene regulation interesting drug targets [2].

A family of proteins known as DNA methyltransferases (DNMTs) are primarily responsible for establishing and maintaining the DNA methylation pattern in cells. In humans, there are three catalytically active isozymes of DNMT that methylate DNA: DNMT3a, DNMT3b, and DNMT1. These enzymes catalyze the transfer of a methyl group from *S*-adenosylmethionine (SAM) to cytosines in CpG dinucleotides [1]. While establishing and maintaining the proper DNA methylation pattern is a complex process, DNMT3a and DNMT3b are associated most closely with *de novo* methylation. DNMT1 on the other hand is the predominate maintenance methyltransferase, as this isoform exhibits a preference for methylation of hemimethylated CpG sites [3]. Inhibition of DNA methyltransferases in cells can reactivate epigenetically silenced genes [4], increasing interest in small molecule inhibitors of DNMTs.

Two types of demethylating agents have been described: nucleoside and non-nucleoside. The first small molecules discovered that caused demethylation in cells were cytidine analogs. Nucleoside analogs, 5-azacytidine (VIDAZA) and 5-aza-2′-deoxycytidine (DACOGEN), have been approved for the treatment of myelodysplastic syndrome and acute myeloid leukemia [2]. However, these nucleoside compounds are not direct DNMT inhibitors. Instead, these molecules have a complex mechanism of action that requires conversion to their triphosphate counterparts and subsequent incorporation into DNA. When encountered by the enzyme, the nitrogen at the 5-position of azacytidine results in formation of a covalent DNMT•DNA complex that is cleared by proteolysis [5]. At high doses, these nucleoside inhibitors are cytotoxic. In addition, instability of the compounds in aqueous solutions and enzymatic degradation of the compounds or their metabolites present challenges [2].

Non-nucleoside DNMT inhibitors offer several potential advantages. The rate of incorporation of nucleoside demethylating agents into DNA varies dramatically depending on cell type. Since DNA incorporation of the drug is required for demethylation to occur, these rate differences lead to variabilities in potency, with cells exhibiting slow incorporation being less sensitive to the drug. In addition, a cells ability to slow DNA incorporation could lead to drug resistance [6]. Nucleoside demethylating agents are not isozyme specific. Once incorporated into DNA, the nucleoside inhibitors will form covalent adducts with any DNMT isozyme encountered [2], leading to depletion of all DNMT isozymes. This is of particular importance as mutations in DNMT3a are associated with poor outcomes in myelodysplastic syndrome and acute myeloid leukemia patients [7, 8], indicating a potential risk of tumorigenesis if DNMT3a is targeted by epigenetic drugs. A direct, isozyme-selective inhibitor, one that does not require incorporation into DNA, could circumvent these challenges.

Several non-nucleoside inhibitors have been described in the literature [2, 9, 10]. This class of inhibitor contains compounds of broad chemical diversity that only share a DNA incorporation-independent mechanism. Many of these molecules have other known biological targets in addition to DNMTs [9]. Several molecules in this class are natural products [10]. This class includes the novel inhibitor SGI-1027, a quinolone derivative, that directly inhibits DNMT1, DNMT3a, and DNMT3b with a SAM-competitive mechanism of action [11], hydralazine, a repurposed hypertension antagonist [12], and many other compounds. Detailed information on the mechanism of action and isozyme specificity is lacking for most of the non-nucleoside inhibitors discovered to date.

In 2014, a high throughput screen aimed at discovering DNMT1 inhibitors first identified laccaic acid A (LCA) as a potent non-nucleoside inhibitor [13]. LCA is a highly substituted anthraquinone natural product from the the red scales of the insect *Kerria lacca*. LCA was shown to be a direct, DNA-competitive inhibitor of DNMT1. The compound exhibits moderate selectivity for DNMT1 over DNMT3a *in vitro*. Treatment of MCF-7 breast cancer cells with LCA results in activation of epigenetically silenced genes; this gene activation is synergistic with 5-azacytidine [14]. In addition, LCA treatment of murine *Rgs6* -/- mouse embryonic fibroblasts reverses DNMT1-dependent oncogenic transformation [15]. Simple anthraquinones, such as anthraquinone 2-carboxylic acid, did not inhibit DNMT1 activity *in vitro* or have any effect on gene expression or proliferative or apoptotic phenotypes in the cell-based experiments [14, 15].

The discovery of 3-nitroflavones as DNMT3a inhibitors [16] coupled with the discovery of LCA as a DNMT1 inhibitor, led to the proposal that flavones, anthraquinones, and related molecules could potentially serve as scaffolds for direct isozyme-selective DNMT inhibitors [14]. Here, we describe a small screen of substituted anthraquinone and anthraquinone-like molecules for their ability to inhibit DNMT1 *in vitro*. Differential scanning fluorimetry (DSF) was used to ensure promising molecules directly interact with the enzyme. Two novel anthraquinone inhibitors were discovered with significantly simpler structures than LCA. These compounds are at least 40-fold less potent than LCA, but retain a DNA-competitive mechanism of action and exhibit a slight preference for DNMT1 over DNMT3a.

## Material and methods

### Materials

All chemicals were purchased from Sigma Aldrich unless specified. LCA was purchased from MicroSource Discovery Systems, Inc.

### DNMT expression and purification

Truncated versions of human DNMT1 were expressed from previously reported vectors [17]. RFTS(-) DNMT1 (amino acids 621–1616) and RFTS(+) DNMT1 (amino acids 351–1616) were expressed in Rosetta 2(DE3)pLysS competent cells (Novagen). Cells were induced with 0.4 mM isopropyl 1-thio-β-D-galactopyranoside (Research Products International) at an optical density of 0.6 at 600 nm and grown for 16–18 hours at 15°C. Cells were pelleted by centrifugation and then resuspended in 20 mM Tris, pH 7.5, 500 mM NaCl, 4 mM β-mercaptoethanol, 5% glycerol and lysed by sonication. Following cell lysis, the soluble lysate was subjected to metal affinity purification utilizing Ni Sepharose 6 Fast Flow resin (GE Life Sciences). Bound protein was eluted in 20 mM Tris, pH 7.5, 500 mM NaCl, 400 mM imidazole, 4 mM β-mercaptoethanol, 5% glycerol and subsequently buffered exchanged into buffer lacking imidazole using Bio-Rad Econo-Pac 10DG desalting columns. Desalted protein was further purified using a HiTrap Heparin HP column (GE Life Sciences). Bound protein was eluted with a linear gradient from 0.25 to 1.5 M NaCl in 20 mM sodium phosphate, pH 7.5, 5% glycerol. The C-terminal catalytic domain of DNMT3a (amino acids 611–912) was expressed and purified as previously reported [13]. All purified DNMTs were concentrated and stored in 50% glycerol at -80°C. All proteins were quantified using A_280_ and calculated extinction coefficients.

### DNA methylation assay

An endonuclease-coupled fluorogenic assay was used to measure DNA methylation [17]. In brief, a hemi-methylated, internally quenched, fluorescent hairpin DNA (Integrated DNA Technologies; S1 Table) is used in assays containing DNMT and the methyl donating co-factor *S*-adenosylmethionine (SAM). Methylation generates the cleavage site for Gla I (SibEnzyme); cleavage releases the 5’-fluorophore from the 3’-quencher and generates a fluorescence signal. Assays were initiated by addition of enzymes to solutions containing both substrates. Assays were conducted in Co-Star black 96 well half-area plates in a BioTek Synergy plate reader using excitation and emission wavelengths of 485 nm and 525 nm, respectively. Fluorescence generation was typically followed for 20 minutes when utilizing RFTS(-) DNMT1 and 60 minutes when utilizing RFTS(+) DNMT1 or DNMT3a.

### Compound screening and hit validation

Screening compounds were identified by searching for compounds with at least 60% similarity to LCA using hit2lead.com. 15 substituted anthraquinones or anthraquinone-like molecules were purchased from ChemBridge Corp, San Diego, CA (S2 Table). The compounds were resuspended in DMSO to a final concentration of 10 mM and aliquoted for storage at -20°C. All compounds were screened for inhibition of RFTS(-) DNMT1 at 10 μM and 50 μM using the DNA methylation assay. Assays (0.1 mL) were conducted in triplicate at 37°C in 10 mM Tris, pH 7.5, 100 mM potassium glutamate, 1 mM MgCl_2_, 1 mM DTT, 0.1 mg/mL BSA (New England BioLabs Inc.), and 5% glycerol. Assays contained 10 nM hairpin oligonucleotide substrate, 10 μM SAM, 1.5 nM RFTS(-) DNMT1, 0.5 U Gla I, and 0.5% DMSO. A matched control containing Gla I in the absence of DNMT1 was subtracted from each assay condition to account for both background fluorescence and slow Gla I cleavage of the internally quenched hairpin DNA substrate. Corrected fluorescence data was averaged and the resulting traces were fitted in KaleidaGraph (Synergy Software) to determine initial velocities. Percent activity was determined by comparing to DMSO-containing control assays.

#### Gla I inhibition assay

Compounds that reduced fluorescence generation in the coupled DNA methylation assay due to inhibition of Gla I were identified and excluded using a Gla I activity assay. Triplicate assays (0.1 mL) were conducted in black 96 well half-area plates in the same buffer used for the DNA methylation assay. Assays containing 20 nM of the internally quenched hairpin DNA substrate were incubated in the presence of 2 nM RFTS(-) DNMT1 and 20 μM SAM for 30 minutes at 37°C in order to generate the substrate for Gla I, the fully methylated hairpin oligonucleotide. Then, 50 μM screening compound, resulting in 0.5% DMSO, was added to each assay and the plate was again incubated at 37°C for 2 minutes. 0.4 U of Gla I or buffer was then added to each well to initiate the reaction and fluorescence generation was followed for 35 minutes. A matched control reaction in the absence of Gla I was subtracted from each assay condition. Corrected fluorescence data was averaged and the resulting time courses were fitted in KaleidaGraph to determine initial velocities. Percent activity was calculated by comparing to a DMSO-containing control assay.

#### Differential scanning fluorimetry assay

Compounds that inhibited fluorescence generation in the DNA methylation assay, but not in the Gla I counter screen, were subjected to differential scanning fluorimetry (DSF) [18]. Assays (25 μL) were conducted in triplicate in a Roche LightCycler 96 Real-Time PCR System. Assays contained 2 μM RFTS(-) DNMT1, 100 μM screening compound, and 5X Sypro Orange (Invitrogen Molecular Probes) in 20 mM HEPES, pH 7.5 and 150 mM NaCl. A DMSO control assay in the absence of compounds was also examined. Fluorescence was measured as temperature was increased from 37 to 85°C in 0.5°C increments. Fluorescence traces were exported, triplicate data was averaged, and the resulting traces were analyzed by fitting to the Boltzmann equation in Prism (GraphPad Software) to determine the observed melting temperature (T_m_).

#### Detergent test

The effect of Triton X-100 on inhibition was examined to exclude compounds that inhibit via aggregation [19]. Assays (0.1 mL) were conducted in triplicate in the DNA methylation assay buffer and contained 10 nM hairpin DNA substrate, 10 μM SAM, 30 μM screening compound, 1.5 nM RFTS(-) DNMT1, and 0.5 U Gla I in the presence and absence of 0.01% Triton X-100. A matched assay lacking DNMT1 was subtracted from each assay condition. Corrected fluorescence data was averaged and initial velocities were determined by linear regression using KaleidaGraph. Percent activity was determined by comparing to a DMSO-containing control assay.

### Determination of IC_50_

The concentration dependence of inhibition was examined for each validated direct DNMT1 inhibitor. IC_50_ values were determined under identical assay conditions (10 nM hairpin DNA substrate and 10 μM SAM) using the DNA methylation assay buffer. Assays (0.1 mL) were conducted in triplicate and contained 1.5 nM RFTS(-) DNMT1, 0.6 U Gla I, 1% DMSO and varied inhibitor concentrations (0–100 μM). A matched control lacking DNMT1 was subtracted from each assay condition. Corrected fluorescence data was averaged and traces were fitted in Kaleidagraph to determine initial velocities. The percent activity at each inhibitor concentration was determined by comparing to a DMSO control assay. Each percent activity reported is an average of at least 3 independent experiments. IC_50_ values were calculated by fitting the percent activity data using a unity Hill slope in KaleidaGraph.

To examine stability of the inhibitors, identical assay solutions were created that contained 100 μM of each compound or DMSO in the DNA methylation assay buffer. Substrates were added to one set of solutions and these solutions were assayed immediately by adding enzymes and observing the change in fluorescence over time. The other set was incubated at room temperature for 60 minutes and then substrates were added. Enzymes were added to initiate these assays and fluorescence changes were monitored. In both cases, assays contained 10 nM hairpin DNA substrate, 10 μM SAM, 1.5 nM RFTS(-) DNMT1, 0.6 U Gla I, and 1% DMSO. A matched control lacking DNMT1 was subtracted from each assay condition. Corrected fluorescence data was averaged and fitted in Kaleidagraph to determine initial velocities. Percent activity was calculated by comparing to a DMSO control assay.

### Mechanism of inhibition

The DNA methylation assay was used to determine the mechanism of inhibition of compound A13. Kinetics assays (0.1 mL) containing 100 μM SAM, 2 nM RFTS(-) DNMT1, 0.8 U Gla I, 0.5% DMSO, and varying amounts of hairpin oligonucleotide substrate (2–25 nM) and compound A13 (0–30 μM) were conducted in triplicate. A matched control lacking DNMT1 was subtracted from each assay condition. Corrected fluorescence data was averaged and traces were fitted in KaleidaGraph to determine initial velocities (in RFU/min). Initial velocities were analyzed globally using nonlinear regression to the competitive inhibition equation in Prism to determine the K_i_.

#### Fluorescence polarization

Fluorescence polarization was used to examine DNA-competitive inhibitor binding. An 18 base pair hemimethylated duplex DNA with a 5’ fluorescein on one strand (Integrated DNA Technologies; S1 Table) was used in binding assays. Assays (50 μL) contained 300 nM RFTS(-) DNMT1, 100 nM fluorophore tagged DNA, and varying concentrations of compound in 20 mM Tris, pH 7.5, 100 mM KCl, 1% DMSO. Assays were conducted in triplicate in black 96 well half-area plates. Following a two-hour incubation at 4°C, the plates were incubated in a BioTek Synergy plate reader equipped with a fluorescence polarization filter cube at 28°C for 30 minutes and then polarization was read using filters with an excitation of 485/20 nm and an emission of 528/20 nm.

### Compound selectivity

Inhibitor selectivity was assessed by examining inhibition of RFTS(+) DNMT1 and the C-terminal catalytic domain of DNMT3a. Assays (0.1 mL) were conducted in triplicate at 37°C and contained 200 nM hairpin oligonucleotide substrate, 0.25 mM SAM, 100 μM compound, 20 nM DNMT, 0.4 U Gla I, and 1% DMSO in the DNA methylation assay buffer. A matched Gla I control assay which did not contain DNMT was subtracted from each reaction trace. Triplicate corrected fluorescence data were averaged and the resulting traces were fitted in Kaleidagraph to determine initial velocities. Percent activity was determined by comparing to a DMSO-containing control.

## Results

### Screening substituted anthraquinones

It has been suggested that substituted anthraquinones and related molecules could serve as a scaffold for direct isozyme-specific DNMT inhibitors [13, 14]. For this reason, we decided to investigate a small set of substituted anthraquinone-like molecules for the ability to inhibit DNMT1, the maintenance methyltransferase. The molecules were selected by searching for screening compounds from ChemBridge Corp. with at least 60% similarity to LCA, a highly substituted anthraquinone natural product; LCA is known to be a direct, DNA-competitive DNMT1 inhibitor [14]. All molecules selected for study contained a polycyclic aromatic core structure, most were anthraquinones, with 1–4 substituents (S2 Table).

**Fig 1:**
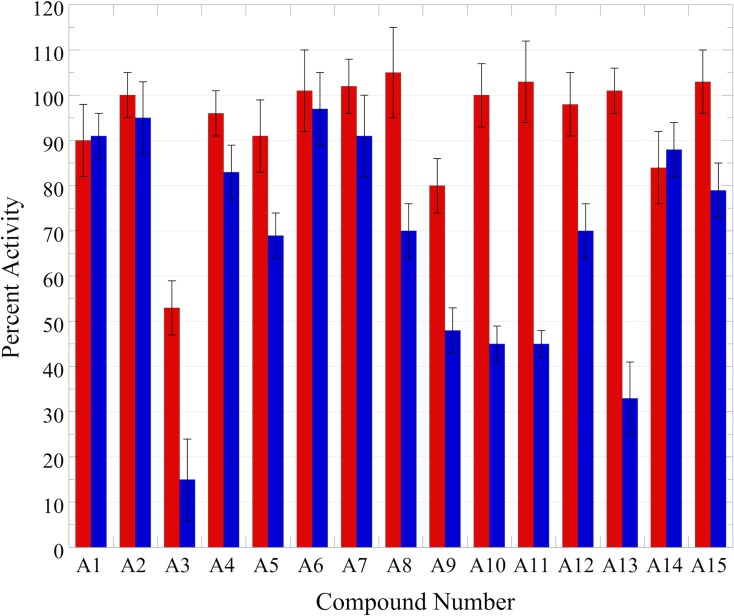
Observed inhibition of RFTS(-) DNMT1 by anthraquinone compounds. Each of the 15 compounds (A1 –A15) were examined at 10 μM (red) and 50 μM (blue) using the endonuclease-coupled DNA methylation assay. The percent activity observed was determined by comparing to a DMSO-containing control assay; all initial velocity data used to generate percent activities is available in S3 Table. At 50 μM, 8 of the 15 compounds reduced activity by at least 30%.

**Fig 2:**
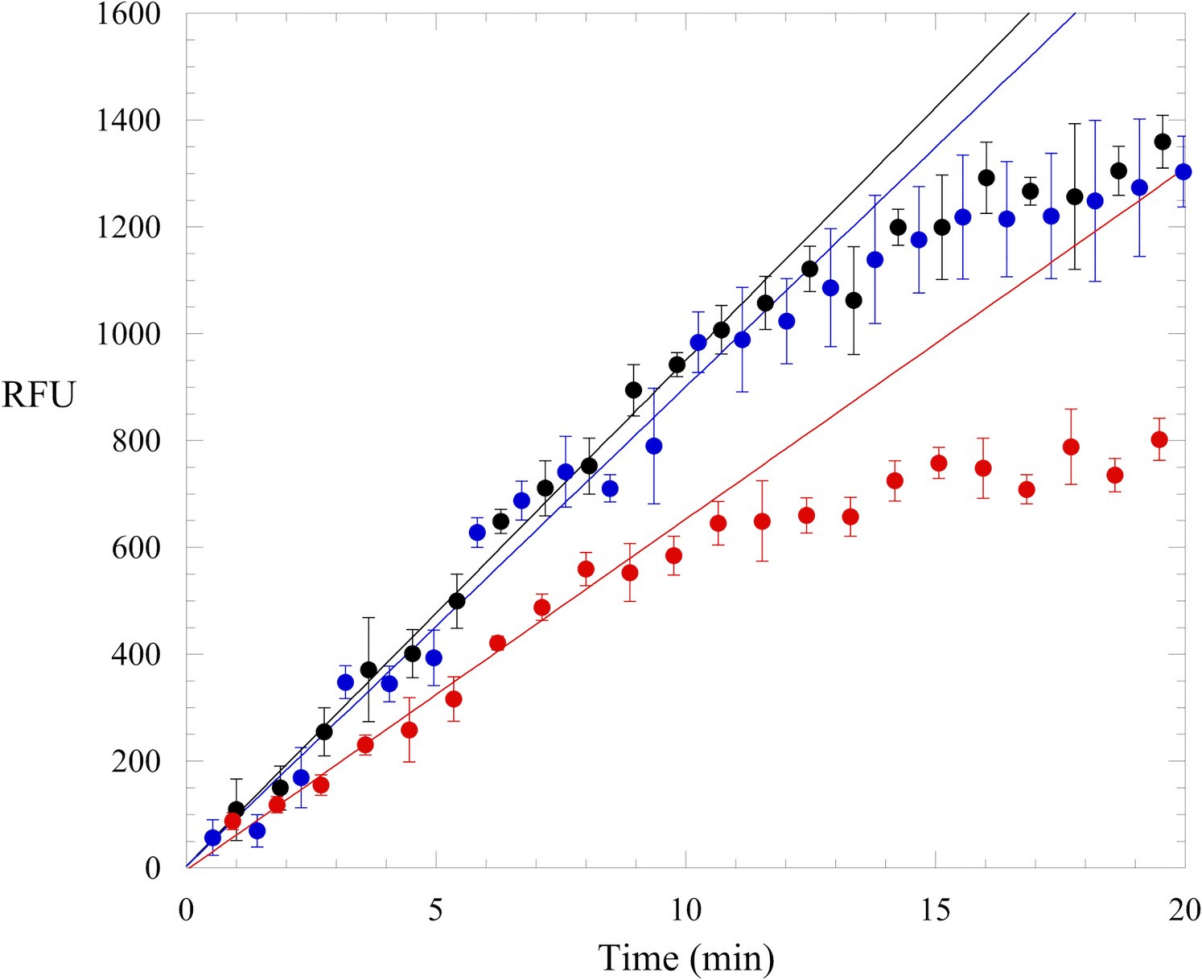
RFTS(-) DNMT1 activity in the endonuclease-coupled DNA methylation assay. Example reactions from the anthraquinone screen containing DMSO (black), 50 μM compound A2 (blue), or 50 μM compound A5 (red). The average and standard error of triplicate assays is shown. Linear regression of the traces gives initial velocities (in RFU/min) of 95 ± 3, 90 ± 6, and 66 ± 4 for reactions containing DMSO, A2, and A5, respectively.

All compounds were screened for DNMT1 inhibition using an endonuclease-coupled DNA methylation assay [13, 17]. Methylation of an internally quenched hemi-methylated hairpin DNA generates the cleavage site for Gla I; cleavage by the endonuclease releases the fluorophore from the quencher and generates fluorescence in real-time. A truncated, activated form of human DNMT1 was used for this screen. Elimination of sequences N-terminal of and including the RFTS domain results in an enzyme with significantly increased catalytic efficiency [17]. The increase in catalytic power of the activated enzyme, referred to as RFTS(-) DNMT1, allows for easier identification of potential inhibitors. In fact, RFTS(-) DNMT1 and the fluorogenic DNA methylation assay have previously been used in a high throughput screen for DNMT inhibitors [13]; this screen first identified the anthraquinone LCA as a DNMT inhibitor. Compounds were initially accessed for RFTS(-) DNMT1 inhibition at 10 μM. At this concentration, addition of only two compounds resulted in DNMT1 activity that was 80% or less than the activity observed in a DMSO-containing control assay (Fig 1; S3 Table). The majority of the compounds examined had no significant effect on observed activity. Since collectively these molecules are significantly simpler in structure than LCA, we decided to screen the compounds again at a higher concentration of 50 μM. Under these conditions, some compounds still had no effect on fluorescence generation in the coupled DNA methylation assay (Fig 2). However, a subset of the compounds examined were able to significantly inhibit fluorescence generation. At 50 μM, 8 of the 15 compounds examined decreased RFTS(-) DNMT1 activity to 70% or less of the activity observed in a DMSO control assay (Fig 1; S3 Table). These 8 molecules were considered preliminary hits and were subjected to further analyses.

Reduction of fluorescence generation in the coupled DNA methylation assay does not necessarily mean the compounds are inhibiting DNMT1 directly. The molecules could be inhibiting the coupling enzyme, Gla I, or potentially interacting with the DNA. To ensure the reduction in observed activity was not due to interference with some aspect of the coupled DNA methylation assay, we examined the ability of each preliminary hit to inhibit fluorescence generation in a Gla I activity assay. Compound A2, which did not inhibit activity in the DNA methylation assay screen (Figs 1 and 2), was examined as a control. Addition of 50 μM A2 to the Gla I activity assay did not affect fluorescence generation (Table 1; S4 Table). Addition of 4 of the preliminary hits resulted in activities ≥88% of the activity observed in a DMSO control assay, indicating that these compounds do not interfere with the assay itself. However, addition of 4 molecules resulted in activities <70% of that observed in the DMSO control assay (Table 1). For this reason, these molecules were not studied further.

**Table 1:** Effect of preliminary hits on Gla I activity.

Compound	Percent Activity
A2	97 ± 6
A3	35 ± 4
A5	68 ± 5
A8	88 ± 10
A9	52 ± 4
A10	51 ± 5
A11	97 ±7
A12	94 ± 9
A13	95 ± 8

LCA is a direct inhibitor of DNMT1 [14]. To ensure the preliminary hits from this screen are also directly interacting with DNMT1, DSF [18] was used to determine observed melting temperatures (T_m_). Compounds that directly interact with DNMT1 stabilize the protein against thermal denaturation, resulting in a right-shift in the observed T_m_. DSF assays were conducted in the absence of substrates. Therefore, a shift in the observed T_m_ indicates that the anthraquinone compound is able to directly bind to the enzyme without either substrate present and protect against thermal denaturation. Addition of LCA resulted in a 2°C right-shift in the observed T_m_ of RFTS(-) DNMT1 (Table 2 and S1 Fig), consistent with previously published work [14]. Addition of two of the preliminary hits investigated also stabilized RFTS(-) DNMT1 against thermal denaturation, shifting the observed T_m_ to the right by roughly 1°C (Table 2 and S1 Fig). The other compounds investigated did not significantly affect the observed T_m_. For this reason, they were not studied further.

**Table 2:** Changes in thermal stability of RFTS(-) DNMT1 in the presence of small molecules.

Compound	Observed T_m_ (°C)	ΔT_m_
DMSO	44.4 ± 0.1	-
A8	44.7 ± 0.1	0.3
A11	45.4 ± 0.1	1.0
A12	44.7 ± 0.1	0.3
A13	45.3 ± 0.1	0.9
LCA	46.4 ± 0.1	2.0

Two of the original 15 compounds examined inhibited fluorescence generation in the coupled DNA methylation assay while not themselves interfering with the Gla I cleavage step required for generation of fluorescence signal and protected the enzyme from thermal denaturation, indicating that these molecules were acting as direct RFTS(-) DNMT1 inhibitors. To ensure the mechanism of inhibition utilized by these apparent inhibitors was not simply aggregation, a detergent test was preformed [19]. Inhibition of A11 and A13 was examined in the presence and absence of 0.01% Triton X-100. Loss of observed inhibition in the presence of detergent indicates that the molecules are inhibiting via nonspecific aggregation of proteins. Addition of detergent had no effect on the inhibition observed with compound A11 or A13 (S5 Table). Thus, all biochemical evidence points to both A11 and A13 being direct inhibitors of RFTS(-) DNMT1.

### Determination of IC_50_ values

**Fig 3:**
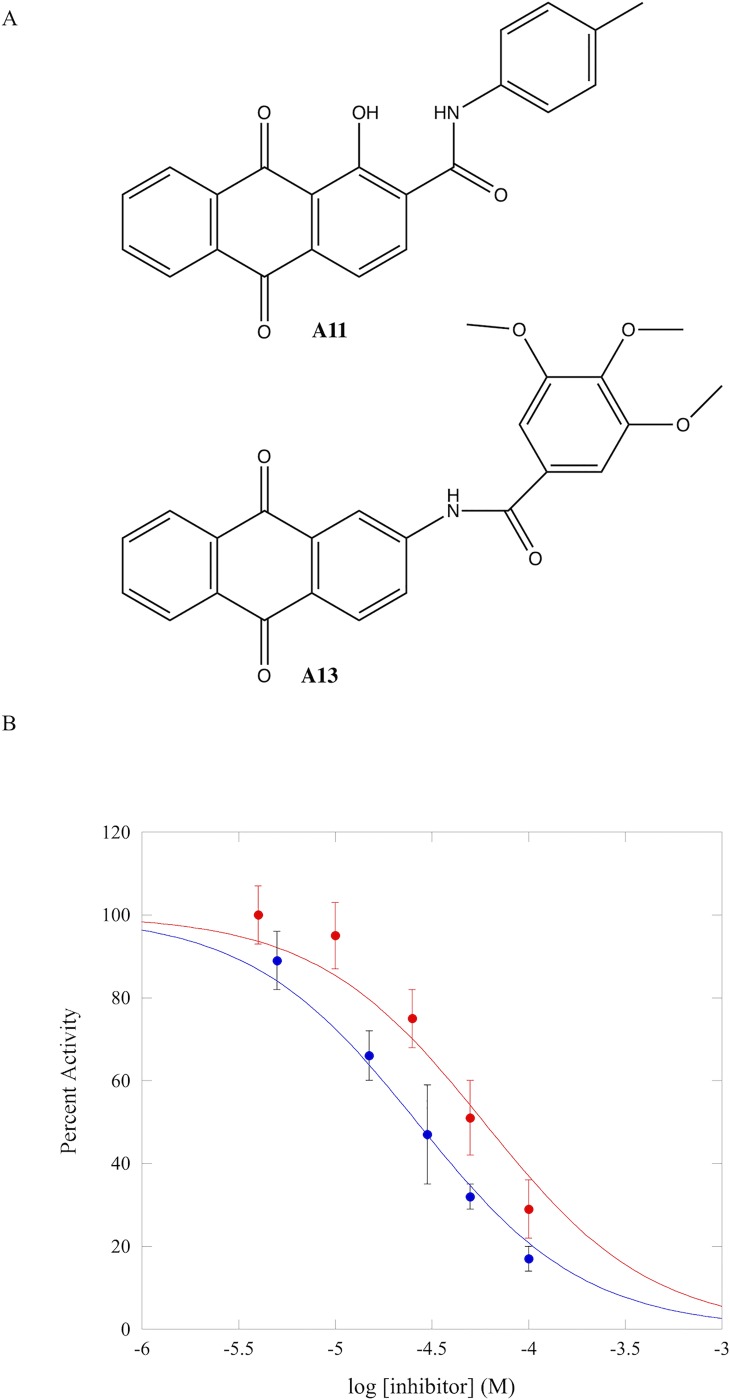
Determination of IC_50_ values. (A) Structures of identified hits A11 and A13. (B) IC_50_ values were determined at 10 nM hairpin DNA substrate and 10 μM SAM for A11 (red) and A13 (blue) using RFTS(-) DNMT1 in the DNA methylation assay. Percent activity at each inhibitor concentration was determined by comparing to a DMSO control assay. Percent activity values reported are the average of at least 3 independent experiments (S6 Table); error bars represent standard deviation. Fitting the data using a unity Hill slope gives IC_50_ values of 59 ± 10 μM for A11 and 26 ± 2 μM for A13.

Our initial biochemical assays indicated that both compound A11 and A13 (Fig 3A) were direct DNMT1 inhibitors; these compounds are both substituted anthraquinones with significantly simpler structures than LCA. To gauge the potency of these molecules, the concentration dependence of inhibition of RFTS(-) DNMT1 was examined under identical conditions (10 nM hairpin DNA substrate and 10 μM SAM) using the DNA methylation assay. The concentration of anthraquinone compound was varied from 0 to 100 μM. Addition of high concentrations of the inhibitors to the assay has no effect on the pH of the solutions. Even at 100 μM compound, the resulting assay solutions had pH values within 0.02 of one another. Both compounds exhibited concentration-dependent inhibition in the assay (Fig 3B and S6 Table). The IC_50_ values observed for A11 and A13 were 59 ± 10 μM and 26 ± 2 μM, respectively. These values are significantly higher than the IC_50_ of LCA. Under similar assay conditions, LCA exhibits an IC_50_ of ~650 nM [14]. To ensure A11 and A13 are stable throughout the course of these experiments, we examined inhibition again at 100 μM. The percent activity observed in assays that were prepared and immediately assayed was the same as the percent activity observed when solutions were allowed to incubate at room temperature for one hour before being assayed (S7 Table). This indicates that the compounds are stable in the assay buffer over the course of these experiments. The higher IC_50_ values observed for the newly identified anthraquinones are presumably due to weaker interactions with the protein.

### Mechanism of inhibition

**Fig 4:**
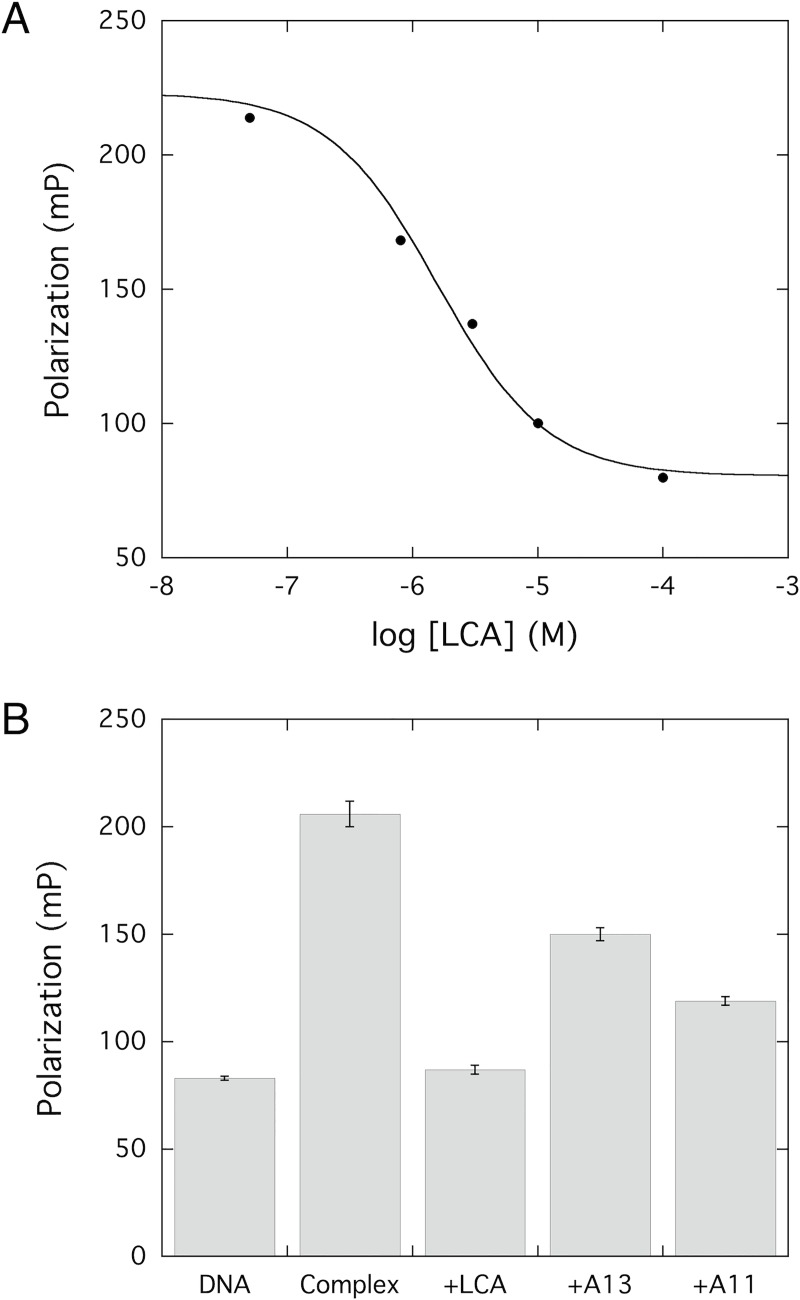
Fluorescence polarization indicates a DNA-competitive mechanism of inhibition. (A) Increasing concentrations of LCA (50 nM– 100 μM) were added to assays containing 300 nM RFTS(-) DNMT1 and 100 nM fluorescein tagged DNA. As LCA concentration was increased, observed polarization values decreased, indicating LCA was competing with DNA for binding to RFTS(-) DNMT1. (B) DNA competition was assessed in the fluorescence polarization assay at 100 μM inhibitor. DNA is the polarization value of free DNA in solution. Complex is the polarization value observed for the RFTS(-) DNMT1•DNA complex. Data points represent the mean and standard deviation of triplicate assays. These data indicate that all three compounds examined are able to compete with DNA for binding to RFTS(-) DNMT1.

LCA is a DNA-competitive inhibitor of DNMT1 [14]. We were interested in seeing if the simpler anthraquinone molecules discovered in this work retain this mode of inhibition. We used fluorescence polarization to begin to explore the DNA-competitive binding mode of the anthraquinone compounds. Free in solution, the fluorescently tagged hemimethylated duplex DNA has a low polarization signal. When bound to RFTS(-) DNMT1, the polarization value increases significantly. If a molecule is capable of competing with DNA for binding to the protein, addition of that molecule should decrease the observed polarization. This is exactly what is observed for LCA, the known DNA-competitive DNMT1 inhibitor (Fig 4A). Addition of increasing concentrations of LCA results in a reduction of the polarization signal. At the highest LCA concentration investigated (100 μM) essentially all the DNA was displaced and the polarization value obtained was the same as the value for DNA free in solution. Since the anthraquinone molecules identified in this study are significantly less potent than LCA, obtaining a complete concentration dependence data set was technically challenging. Instead, we examined competition at a signal inhibitor concentration (100 μM). Under these conditions, free DNA had a polarization signal of 83 ± 3 mP. Addition of RFTS(-) DNMT1 resulted in an increase in polarization to 206 ± 6 mP, indicating that the DNA was bound to the protein. Adding LCA to this complex freed the bound DNA resulting in a polarization value of 87 ± 2 mP (Fig 4B; S8 Table). Addition of A13 and A11 also decreased the observed polarization signal to 150 ± 3 and 119 ± 2 respectively (Fig 4B; S8 Table). Addition of the newly identified compounds did reduce the observed polarization value, indicating that they are both capable of competing with DNA for binding to RFTS(-) DNMT1, however, neither compound was as effective of an inhibitor as LCA.

**Fig 5:**
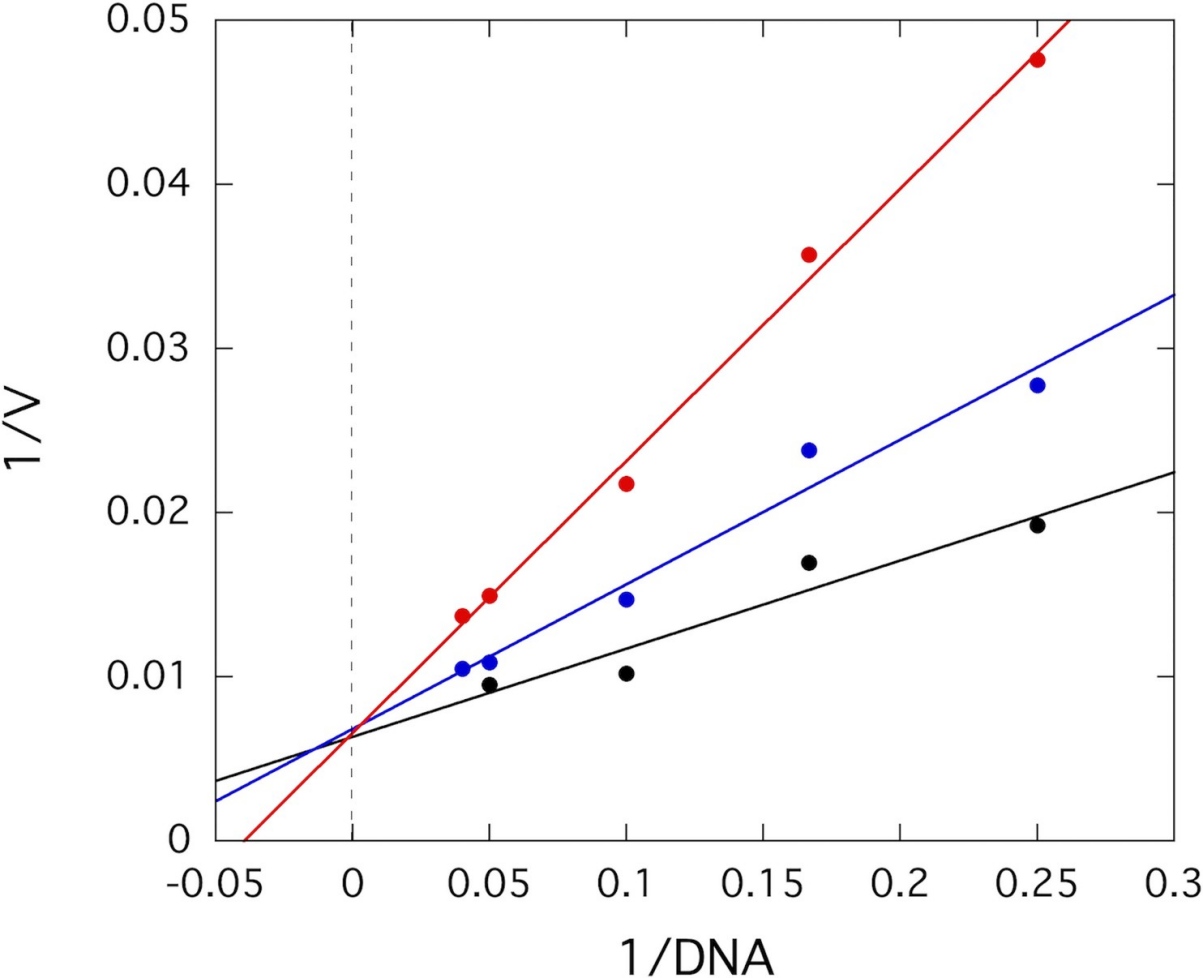
A13 is a DNA-competitive inhibitor of RFTS(-) DNMT1. A double reciprocal plot of DNA-dependent A13 inhibition kinetics is shown. A13 was used as an inhibitor in reactions containing 100 μM SAM and varying concentrations of DNA (2–25 nM). The velocity data without A13 (black), with 15 μM A13 (blue), and with 30 μM A13 (red) are indicative of competitive inhibition. Fitting the data to a competitive inhibition model gives a K_i_ value of 13 ± 3 μM.

We then decided to investigate the mechanism of inhibition more thoroughly using kinetics. We examined only compound A13, since this molecule exhibited a ~2-fold higher potency in the IC_50_ experiments. We titrated A13 in assays containing high concentrations of SAM and varied concentrations of DNA in order to determine kinetic parameters. The double reciprocal plot of the data clearly shows competitive inhibition with all lines intersecting on the y-axis. As the concentration of A13 was increased, the apparent K_m,DNA_ was increased with no effect on the observed V_max_ (Fig 5 and S2 Fig). Fitting these data by nonlinear regression to a competitive inhibition model, a K_i_ of 13 ± 3 μM was obtained.

### Selectivity of anthraquinone inhibitors

Three catalytically active isozymes of human DNMT exist: DNMT3a, DNMT3b, and DNMT1 [1]. LCA shows a preference for DNMT1 inhibition [13, 14]. We wanted to examine whether these simplified anthraquinones inhibitors retained this isozyme selectivity. We have shown that the simpler compounds identified here are direct, DNA-competitive inhibitors of the truncated activated form of DNMT1 (RFTS(-) DNMT1). In order to more accurately assess isozyme selectivity, RFTS(+) DNMT1 (amino acids 351–1616) was used. This version of the protein contains the inhibitory RFTS domain and behaves like full-length DNMT1 in *in vitro* assays [17]. In addition, the C-terminal catalytic domain of DNMT3a was used. Employing the endonuclease-coupled DNA methylation assay, the percent activity observed for each methyltransferase was determined at 100 μM inhibitor. We chose to examine inhibition at 100 μM inhibitor because the simpler anthraquinone compounds identified here are weaker DNMT1 inhibitors than LCA. In addition, the RFTS domain itself is a DNA-competitive inhibitor of DNMT1 activity [17], so higher concentrations of DNA-competitive inhibitors are required to displace the endogenous inhibitory protein domain when present. Compound A6, which did not inhibit the activity of RFTS(-) DNMT1 in the original screen (see Fig 1), was examined as a control. As expected, addition of A6 did not affect the activity of either methyltransferase (Table 3 and S9 Table). We also examined inhibition by LCA. The percent activity observed for RFTS(+) DNMT1 and DNMT3a were both fairly low (<32%). This is in line with previously published results [14]. Under these assay conditions (200 nM DNA and 0.25 mM SAM), 100 μM LCA is above the observed IC_50_ value for both enzymes, causing the selectivity to be obscured. Both newly identified molecules showed a slight preference for DNMT1 under these conditions (Table 3 and S9 Table). Interestingly, compound A13 did not inhibit DNMT3a activity at all under these conditions, but was able to inhibit RFTS(+) DNMT1 activity by nearly 50%.

**Table 3:** Percent activity observed with different DNMT isozymes.

Compound	RFTS(+) DNMT1	DNMT3a catalytic domain
A11	65 ± 3	83 ± 2
A13	58 ± 2	103 ± 3
A6	93 ± 4	95 ± 3
LCA	21 ± 1	32 ± 1

## Discussion

Changes to the methylation pattern in cells has been linked to the initiation and progression of cancer. Targeting these epigenetic changes is a potential cancer therapeutic strategy, making the enzymes responsible for DNA methylation in cells drug targets. While some nucleoside inhibitors like 5-azacytidine are approved drugs, these molecules have many pitfalls [2]. These demethylating compounds are not isozyme specific DNMT inhibitors and instead require incorporation into DNA to exert their effects [5]. These compounds act non-specifically on the genome, causing the activation of several genes and potentially transposons, which can lead to genome instability [20, 21]. In addition, nucleoside analogs can be incorporated into RNA and induce off-target effects [5]. It clearly seems like there is a need for the discovery of new inhibitors that can overcome these pitfalls and reduce side effects in patients.

Direct, isozyme specific inhibitors could be advantageous. In addition to serving as the starting point in future drug development efforts, a compound targeted at DNMT1 enzymatic activity could be useful in furthering our understanding of cancer etiology. Many current genetic approaches rely on reduction or complete loss of protein. For example, reducing DNMT1 activity using hypomorphic alleles protects against cancer formation in *Apc*^*Min*^ mice [22]. Tumorigenesis was also inhibited in cell culture systems and mouse tumor models by targeting *Dnmt1* with antisense oligonucleotides [23, 24]. These approaches are useful to distinguish the functions of the various isozymes of DNMTs. However, loss of protein experiments cannot easily distinguish the importance of DNA methylation activity from DNMT physical interactions in gene silencing and carcinogenesis. Small molecules that specifically block enzyme activity or inhibit a specific protein interaction could perform differently than genetic experiments that prevent protein expression. This could be important for fully understanding the function of a large multi-domain protein such as DNMT1, which is known to be involved in several regulatory interactions [25, 26].

Thus, direct, isozyme specific small molecules could be useful molecular probes as well as potential leads for development of novel cancer therapeutics. Here, we screened a small set of anthraquinone-like molecules for direct inhibition of DNMT1 activity. We chose to investigate substituted anthraquinones due to the recent discovery of LCA, a highly substituted anthraquinone natural product, as a direct DNMT1 inhibitor [13, 14]. LCA was first discovered in a high throughput screen of the Spectrum compound collection. In this screen, compounds were assessed for inhibition at ~11 μM [13]. When a similar assay was used to examine RFTS(-) DNMT1 inhibition with this set of compounds, essentially no inhibition was observed at 10 μM compound. The compounds examined here are significantly simpler in structure than LCA, which contains seven substituents around the anthraquinone core structure; LCA contains four hydroxyl groups, two carboxyl groups, and a large aromatic ring containing substituent (N-acetyltyramine) (S3 Fig). The compounds examined in this work only contained 1–4 substituents around the polycyclic core structure, with most compounds containing only one or two substituents. One would expect the loss of potential interactions with the protein due to the simplified structures of the screening compounds to result in compounds with decreased potency as compared to LCA. Screening the compounds for RFTS(-) DNMT1 inhibition again at a higher concentration, 50 μM, resulted in eight initial hits. Interestingly, all the initial hits identified are substituted anthraquinones. This set of screening compounds did contain three molecules which were not anthraquinones; for example, a couple substituted fluorenes were examined. None of the non-anthraquinone compounds assayed inhibited RFTS(-) DNMT1 in our assays.

While LCA contains several substituents, it contains one larger aromatic substituent. N-acetyltyramine is bound directly to the carbon in the 2-position of the anthraquinone core ortho to its hydroxyl (S3 Fig). Eight of the compounds we screened for DNMT1 inhibition contained large substituents at C2 of the anthraquinone core. Four screening compounds were anthraquinones with either a small C2 substituent, like a hydroxyl or carboxyl group, or no C2 substituent. Interestingly, most of the anthraquinones identified as initial hits in our screen (6 of 8) contain a larger substituent at the 2-position of the anthraquinone core. In five of the initial hits, the substituent at C2 contains an aromatic ring. Previous work showed that 9,10-anthraquinone and anthraquinone 2-carboxylic acid are not inhibitors of RFTS(-) DNMT1 *in vitro* [14]. Taken together, this indicates that a larger C2 substituent on the anthraquinone core could be important for DNMT1 inhibition.

Of the initial eight hits from our screen, four compounds inhibited Gla I activity, the coupling enzyme used in the DNA methylation assay, in control assays. This does not preclude these molecules from being true inhibitors of DNMT1. However, interfering with our DNA methylation assay made these molecules difficult to study further. These compounds could be examined for DNMT1 inhibition using an assay that does not require the endonuclease Gla I. For example, radioactivity has been successfully used for decades to study the activity of DNA methyltransferases [27, 28]. Examining inhibition by these molecules using a different DNA methylation assay could very well show that the compounds are bonafide DNMT1 inhibitors.

Interestingly, the four compounds that inhibited RFTS(-) DNMT1 activity and did not interfere with our methylation assay were all anthraquinones with large C2 substituents. In three of these molecules, the substituent contained an aromatic ring. The fourth compound contained a linear substituent at C2. Only two of these compounds stabilized thermal denaturation of RFTS(-) DNMT1 by at least 0.9°C, indicating that these small molecules were directly interacting with the enzyme. This temperature shift cutoff is in line with previous studies that have shown changes in observed T_m_ values of 1–2°C or greater for direct binders of proteins [13, 18, 29, 30]. Our differential scanning fluorimetry experiments were conducted at 100 μM compound. We were unable to screen for direct binding at higher inhibitor concentrations due to the interference of higher concentrations of DMSO on the DSF assay. The two compounds that did not significantly shift the observed melting temperature in the assay, A8 and A12, are presumably weaker inhibitors of RFTS(-) DNMT1 than the other two compounds. In our initial RFTS(-) DNMT1 activity screen, addition of 50 μM of compound A11 or A13 decreased observed activity by more than 50%. However, addition of compound A8 or A12 only decreased activity by 30%. If A8 or A12 are direct inhibitors of RFTS(-) DNMT1, they could require concentrations greater than 100 μM compound to generate enough of the enzyme•inhibitor complex to observe stabilization of thermal denaturation. This is an interesting finding for compound A12, which is an isomer of one of our verified hits A11; the only difference in the structures is the position of the methyl group on the aromatic ring of the C2 substituent. In A11, the methyl group is para to the nitrogen in the amide linker (Fig 3A). However, in A12, the methyl group is ortho to the nitrogen in the amide linker. The movement in position of this methyl group presumably weakens association with the enzyme in compound A12.

After all primary and secondary screening, 2 of the 15 compounds examined were found to be direct inhibitors of RFTS(-) DNMT1 enzymatic activity. Both of these compounds have an aromatic-ring containing C2 substituent (Fig 3A). In LCA, the aromatic ring of the large substituent is directly bound to the C2 position of the anthraquinone core (S3 Fig). In our identified inhibitors there is a two atom spacer between C2 of the anthraquinone and the aromatic rings in the substituent (Fig 3A). In both compounds this spacer is an amide. However, the orientation of the amide bond is reversed in the two compounds, indicating that it is the distance that is important for DNMT1 inhibition, not the placement of the nitrogen and carbonyl atoms.

Even with significantly simpler structures, both of the newly identified anthraquinone inhibitors retain the DNA-competitive mechanism of action of LCA. The fluorescence polarization data suggest that both compounds are capable of competing with DNA for binding to RFTS(-) DNMT1. Full competitive binding curves could not be obtained for the newly identified inhibitors using the fluorescence polarization assay. At concentrations of inhibitor greater than 100 μM, DMSO began to affect the polarization values, making it difficult to determine how the small molecules were impacting probe DNA binding. To confirm our fluorescence polarization data, we performed inhibition kinetics studies with A13 to determine if this molecule was DNA competitive. The kinetics clearly indicate a competitive mechanism. The K_i_ obtained for A13 (~13 μM) is significantly larger than the previously reported K_i_ for LCA (~0.3 μM) [14]. This is not unexpected, as the structures of the newly identified inhibitors are significantly less complex than the structure of LCA. Removing potential hydrogen bond donors and acceptors would be expected to result in weaker association.

LCA shows a preference for inhibiting DNMT1 [14]. The isozyme selectivity of LCA is not well understood. No crystal structure of DNMT1 with LCA bound is available. In addition, to our knowledge, no docking or computer simulation studies have been done to examine the potential binding site for LCA. Interestingly, the inhibitors identified in this study also exhibited modest selectivity for DNMT1 over DNMT3a. Retaining some isozyme selectivity with the simplified anthraquinones was a bit of a shock. Alizarin, an anthraquinone with hydroxyls at both the 1- and 2-positions of the anthraquinone core, was also identified as a DNMT1 inhibitor in the screen that identified LCA. Alizarin was less potent than LCA and exhibited no isozyme selectivity [13]. The ability of the simplified anthraquinones identified here to selectively inhibit DNMT1 over DNMT3a suggests that a large C2 substituent on the anthraquinone core is sufficient for observing at least some isozyme selectivity in these substituted anthraquinones. A more detailed understanding of the molecular interactions critical for binding LCA and the newly identified anthraquinone inhibitors is required to fully understand the observed isozyme selectivity.

Taken together, the findings of this work indicate that substituted anthraquinones could serve as a scaffold for developing novel DNMT1 specific inhibitors. The simplified anthraquinones inhibitors, A11 and A13, could serve as a starting point for continued development of DNMT1 inhibitors. Even with simplified structures, these molecules retain the properties observed for LCA [14], the highly substituted anthraquinone natural product. The complex structure of LCA makes synthesis of this molecule difficult. Creating novel compounds starting from the simplified anthraquinone structures identified in this work might be an easier route to developing DNMT1-specific inhibitors. Systematically making changes to the C2 substituent on the anthraquinone core of A11 and A13 could reveal important structural determinants for DNMT1 inhibition. In addition, adding back substituents at key places around the core anthraquinone structure could increase both potency and selectivity of future inhibitors.

Further development of substituted anthraquinones would be aided by structural characterization of these molecules bound to DNMT1. In the absence of crystallographic data, docking studies could provide insight into the potential binding site for LCA and other substituted anthraquinones. Site-directed mutagenesis could be used to verify the importance of particular residues in inhibitor binding. Structure activity relationship data would also be extremely useful. The set of molecules we examined here shared some structural similarities. However, the placement of substituents around the anthraquinone core structure and systematic structural changes to the substituents themselves was not examined in this set of molecules. Series of structurally related novel substituted anthraquinones could be assessed for DNMT1 inhibition *in vitro* or a virtual screening approach could be utilized [31] for future inhibitor development. Substituted anthraquinones have the potential to serve as a rich scaffold for developing DNA-competitive DNMT1-specific inhibitors for use as molecular probes to improve our understanding of the enzymology of DNMT1 in cells and as lead compounds for the generation of novel therapy agents.

## Supporting information

S1 Fig Impact of small molecules on RFTS(-) DNMT1 stability. DSF was used to determine the observed T_m_ of RFTS(-) DNMT1 in the presence of DMSO (black), or 100 μM of A8 (blue), A11 (green), A12 (red), A13 (purple), and LCA (orange). Triplicate assays were averaged to generated observed melting traces; fluorescence traces were normalized. Fitting the observed melting traces to the Boltzmann equation gives observed T_m_ values. Addition of LCA stabilized denaturation by 2°C. Of the new compounds examined, addition of only A11 and A13 shifted the T_m_ to the right, each by roughly one degree.
(TIF)

S2 Fig Mode of inhibition of A13. The inhibition of RFTS(-) DNMT1 by compound A13 was examined kinetically. A13 was used as an inhibitor in reactions containing 100 μM SAM and varying concentrations of DNA (2–25 nM). Triplicate corrected fluorescence data was averaged and fit to determine the initial velocity of each condition. The velocity data without A13 (black), with 15 μM A13 (blue), and with 30 μM A13 (red) are indicative of competitive inhibition. Fitting this data by nonlinear regression to a competitive inhibition model gives a K_i_ value of 13 ± 3 μM.
(TIF)

S3 Fig Structure of Laccaic Acid A. Structure of the known DNA-competitive DNMT1 inhibitor laccaic acid A (LCA). LCA is a highly substituted anthraquinone containing four hydroxyl groups, two carboxylic acids, and one large aromatic ring containing substituent (N-acetyltyramine).
(TIFF)

S1 Table DNA oligonucleotides used in this study. All oligonucleotides were synthesized by Integrated DNA Technologies, Inc.
(DOCX)

S2 Table Compounds screened for DNMT1 inhibition. Screening compounds were identified by searching for molecules with at least 60% similarity to LCA using hit2lead.com. All compounds were purchased from ChemBridge Corp.
(DOCX)

S3 Table Initial velocity data from anthraquinone screen. 15 compounds (A1 –A15) were screened for inhibition of RFTS(-) DNMT1 at 10 μM and 50 μM. In all cases, reactions were conducted in triplicate. A matched reaction in the absence of DNMT1 was subtracted from each assay. The resulting corrected fluorescence data was averaged and fitted in Kaleidagraph to determine the initial velocity. Compounds were assayed in batches. Reported below are initial velocities for each condition (errors are from linear regression). To calculate percent activity, the initial velocity in the presence of inhibitor was divided by the initial velocity observed in the absence of inhibitor; errors from initial velocities were propagated. These percent activities are reported in Fig 1.
(DOCX)

S4 Table Initial velocity data from Gla I counter screen. Compounds that inhibited fluorescence generation in the initial DNMT1 screen, were examined for their ability to inhibit the coupling enzyme used in the assay, Gla I. Reactions, conducted in triplicate, contained 50 μM compound. A matched reaction in the absence of Gla I was subtracted from each assay. The resulting corrected fluorescence data was averaged and fitted in Kaleidagraph to determine the initial velocity. Compounds were assayed in batches. Reported below are initial velocities for each condition (errors are from linear regression). To calculate percent activity, the initial velocity in the presence of inhibitor was divided by the initial velocity observed in the absence of inhibitor; errors from initial velocities were propagated. These percent activities are reported in Table 1.
(DOCX)

S5 Table Effect of Triton X-100 on observed inhibition of RFTS(-) DNMT1. Inhibition by compounds A11 and A13 was observed in the presence and absence of 0.01% Triton X-100 in the endonuclease-coupled DNA methylation assay. Triplicate corrected fluorescence data was averaged and fitted in Kaleidagraph to determine the initial velocity; error is from linear regression. Percent activity was determined by comparing to a DMSO-containing control assay.
(DOCX)

S6 Table IC_50_ determination. The concentration dependence of inhibition by compounds A11 and A13 was determined using the endonuclease-coupled DNA methylation assay. The concentration of inhibitor was varied from 0–100 μM. In all cases, reactions were conducted in triplicate. A matched reaction in the absence of RFTS(-) DNMT1 was subtracted from each assay. The resulting corrected fluorescence data was averaged and fitted in Kaleidagraph to determine the initial velocity. Percent activity was determined by comparing the initial velocity in the presence of inhibitor to the initial velocity observed in the absence of inhibitor. Percent activity from at least 3 independent experiments was averaged; standard deviation was used to calculate the error. Average percent activity data are plotted in Fig 3B.
(DOCX)

S7 Table Examining the stability of A11 and A13. Inhibition of RFTS(-) DNMT1 by compounds A11 and A13 was assessed at 100 μM in the endonuclease-coupled DNA methylation assay. One set of assay solutions was immediately examined. Another set was allowed to incubate at room temperature for 60 minutes before addition of enzymes. In all cases, assays were conducted in triplicate and a matched reaction in the absence of DNMT1 was subtracted from each assay. Triplicate corrected fluorescence data was averaged and fitted in Kaleidagraph to determine the initial velocity. Percent activity was determined by comparing to the DMSO-containing control assay.
(DOCX)

S8 Table DNA-competitive binding using fluorescence polarization. Fluorescence polarization was used to investigate the mechanism of inhibition by compounds A11 and A13. When the fluorophore-tagged DNA is bound by RFTS(-) DNMT1, the polarization value increases. Small molecules capable of competing with DNA for binding should decrease the observed polarization. Assays were conducted in triplicate. The average and standard deviation of the triplicate data is plotted in Fig 4B.
(DOCX)

S9 Table Isozyme specificity of A11 and A13 inhibition. Isozyme specificity was investigated using RFTS(+) DNMT1 and the C-terminal catalytic domain of DNMT3a. Activity of both enzymes was investigated using the endonuclease-coupled DNA methylation assay in the presence and absence of 100 μM inhibitor. In all cases, reactions were conducted in triplicate. A matched reaction in the absence of methyltransferase was subtracted from each assay. The resulting corrected fluorescence data was averaged and fitted in Kaleidagraph to determine the initial velocity. Compounds were assayed in batches. Reported below are initial velocities for each condition (errors are from linear regression). To calculate percent activity, the initial velocity in the presence of inhibitor was divided by the initial velocity observed in the absence of inhibitor; errors from initial velocities were propagated. These percent activities are reported in Table 3.
(DOCX)
