# Supplementary figures and images for: Substituted anthraquinones represent a potential scaffold for DNA methyltransferase 1-specific inhibitors
Source: PLoS One. 2019 Jul 15;14(7):e0219830. doi: 10.1371/journal.pone.0219830 (PMC6629088; doi:10.1371/journal.pone.0219830)

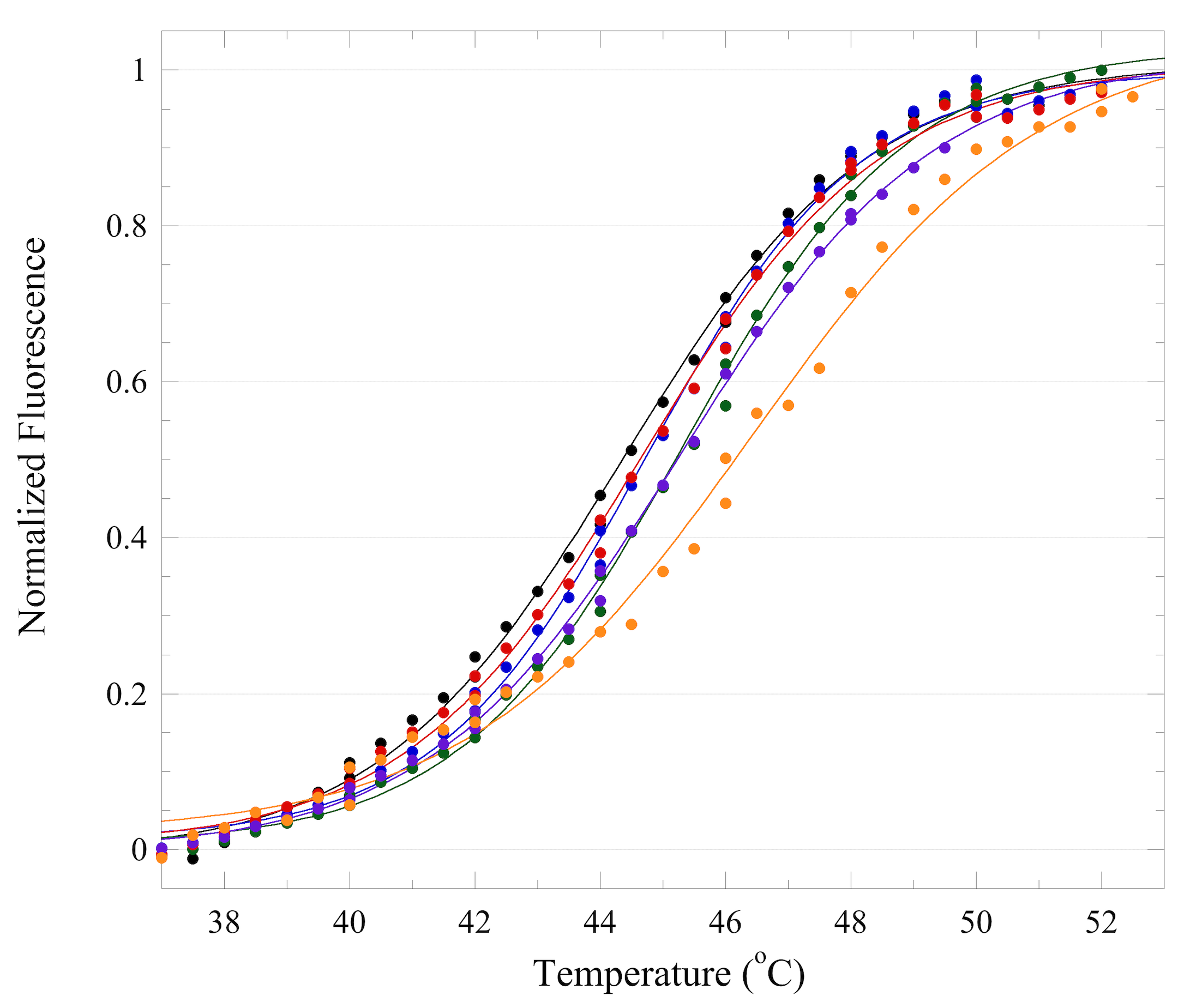

Supplement: S1 Fig — DSF was used to determine the observed Tm of RFTS(-) DNMT1 in the presence of DMSO (black), or 100 μM of A8 (blue), A11 (green), A12 (red), A13 (purple), and LCA (orange). Triplicate assays were averaged to generated observed melting traces; fluorescence traces were normalized. Fitting the observed melting traces to the Boltzmann equation gives observed Tm values. Addition of LCA stabilized denaturation by 2°C. Of the new compounds examined, addition of only A11 and A13 shifted the Tm to the right, each by roughly one degree. (TIF) [file pone.0219830.s001.tif]

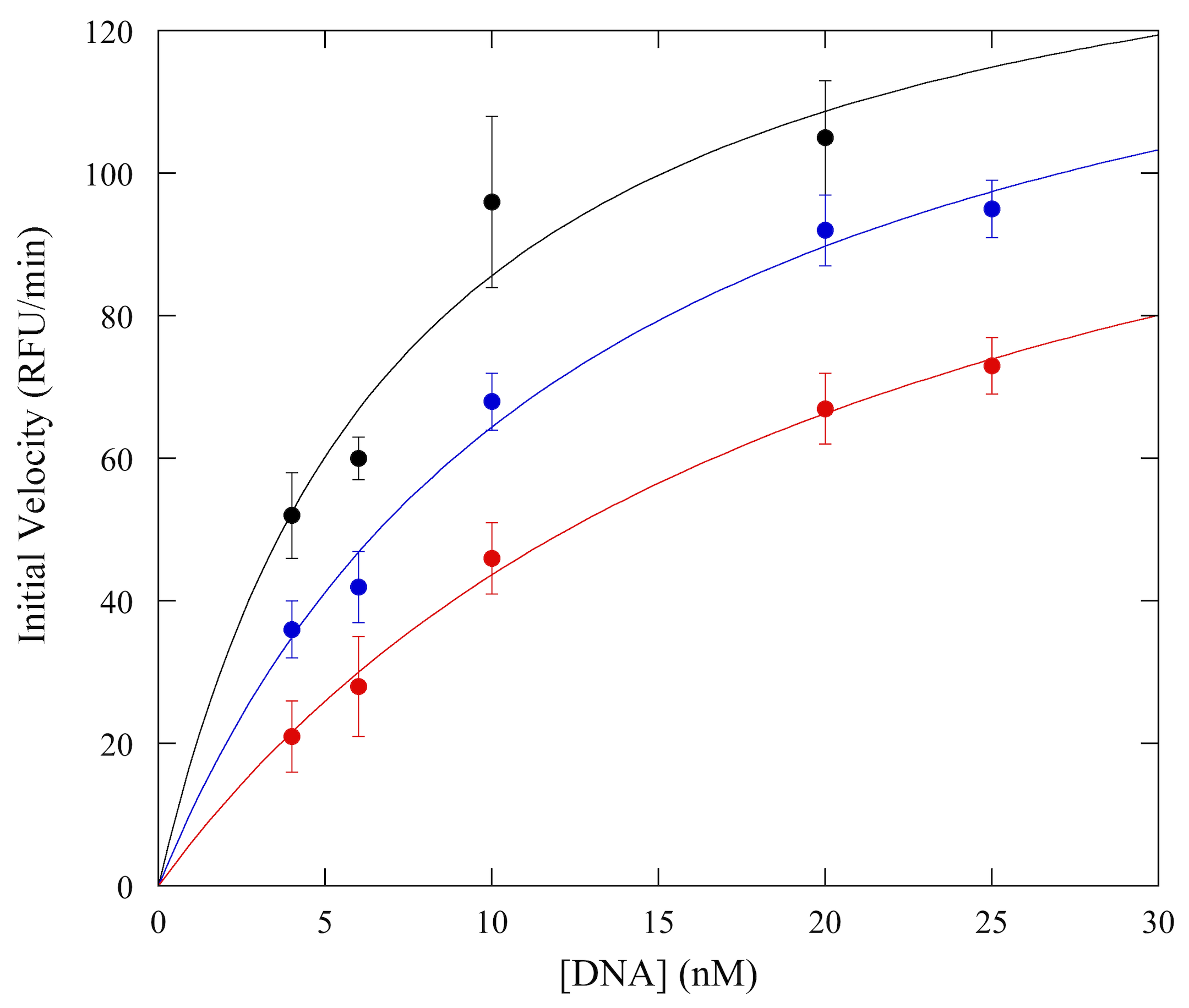

Supplement: S2 Fig — The inhibition of RFTS(-) DNMT1 by compound A13 was examined kinetically. A13 was used as an inhibitor in reactions containing 100 μM SAM and varying concentrations of DNA (2–25 nM). Triplicate corrected fluorescence data was averaged and fit to determine the initial velocity of each condition. The velocity data without A13 (black), with 15 μM A13 (blue), and with 30 μM A13 (red) are indicative of competitive inhibition. Fitting this data by nonlinear regression to a competitive inhibition model gives a Ki value of 13 ± 3 μM. (TIF) [file pone.0219830.s002.tif]

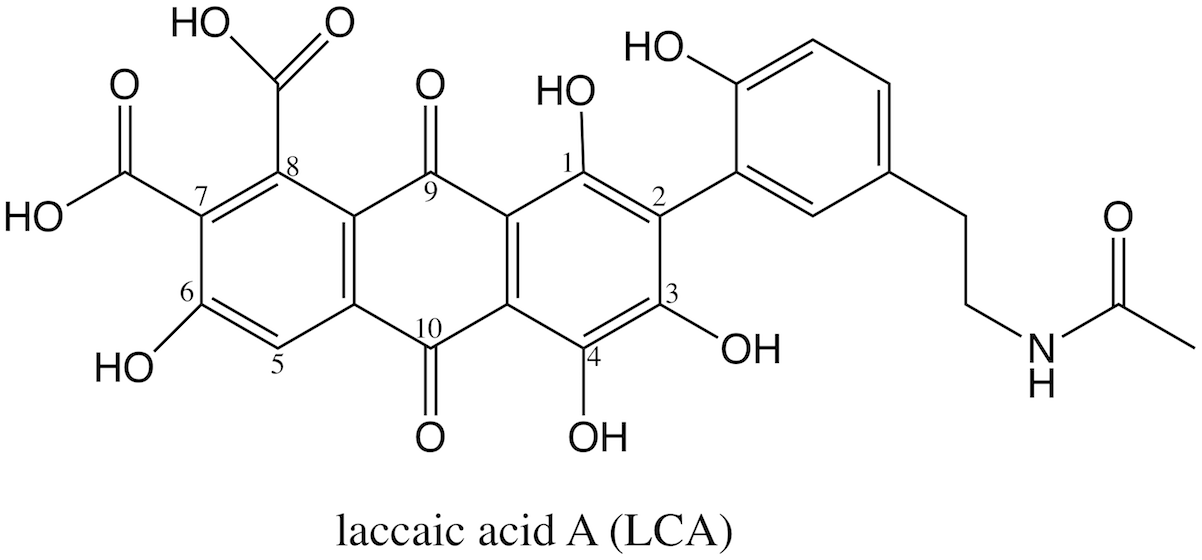

Supplement: S3 Fig — Structure of the known DNA-competitive DNMT1 inhibitor laccaic acid A (LCA). LCA is a highly substituted anthraquinone containing four hydroxyl groups, two carboxylic acids, and one large aromatic ring containing substituent (N-acetyltyramine). (TIFF) [file pone.0219830.s003.tiff]
