# Supplementary material for: Substituted anthraquinones represent a potential scaffold for DNA methyltransferase 1-specific inhibitors
Source: PLoS One. 2019 Jul 15;14(7):e0219830. doi: 10.1371/journal.pone.0219830 (PMC6629088; doi:10.1371/journal.pone.0219830)
Supplement: S1 Table — All oligonucleotides were synthesized by Integrated DNA Technologies, Inc. (DOCX) [file pone.0219830.s004.docx]

**S1 Table. DNA oligonucleotides used in this study.** All oligonucleotides were synthesized by Integrated DNA Technologies, Inc.

| Hairpin DNA for methylation assays | **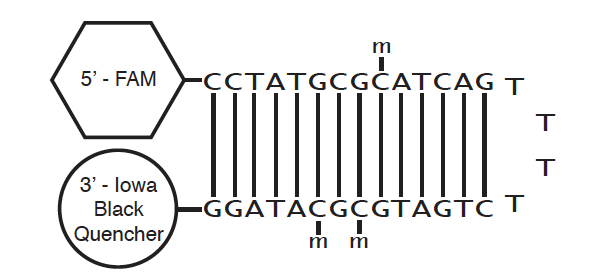** |
| --- | --- |
| 18 bp duplex for fluorescence polarization | **** |
