# Supplementary material for: Substituted anthraquinones represent a potential scaffold for DNA methyltransferase 1-specific inhibitors
Source: PLoS One. 2019 Jul 15;14(7):e0219830. doi: 10.1371/journal.pone.0219830 (PMC6629088; doi:10.1371/journal.pone.0219830)
Supplement: S2 Table — Screening compounds were identified by searching for molecules with at least 60% similarity to LCA using hit2lead.com. All compounds were purchased from ChemBridge Corp. (DOCX) [file pone.0219830.s005.docx]

**S2 Table. Compounds screened for DNMT1 inhibition.** Screening compounds were identified by searching for molecules with at least 60% similarity to LCA using hit2lead.com. All compounds were purchased from ChemBridge Corp.

| Compound | ChemBridge ID # | Compound Name |
| --- | --- | --- |
| A1 | 5228205 | methyl N-(4-hydroxy-9,10-dioxo-9,10-dihydro-1-anthracenyl)-beta-alaninate |
| A2 | 5105559 | 9,10-dioxo-9,10-dihydroanthracene-1,8-diyl diacetate |
| A3 | 5122149 | 1,2,5,8-tetrahydroxyanthra-9,10-quinone |
| A4 | 5174217 | N-9H-fluoren-2-ylbenzamide |
| A5 | 5228189 | 1,4-diamino-N-(4-butylphenyl)-9,10-dioxo-9,10-dihydro-2-anthracenecarboxamide |
| A6 | 5249376 | 1,4-dihydroxy-2-phenoxyanthra-9,10-quinone |
| A7 | 5249405 | 1,4-dihydroxy-2-[(4-methylphenyl)amino]anthra-9,10-quinone |
| A8 | 5249429 | 1-amino-N-(3-methoxypropyl)-9,10-dioxo-9,10-dihydro-2-anthracenecarboxamide |
| A9 | 5474467 | 1-(ethylamino)-9,10-dioxo-9,10-dihydro-2-anthracenecarboxylic acid |
| A10 | 5811179 | 1-hydroxy-N-(3-methoxyphenyl)-9,10-dioxo-9,10-dihydro-2-anthracenecarboxamide |
| A11 | 5812086 | 1-hydroxy-N-(4-methylphenyl)-9,10-dioxo-9,10-dihydro-2-anthracenecarboxamide |
| A12 | 5813388 | 1-hydroxy-N-(2-methylphenyl)-9,10-dioxo-9,10-dihydro-2-anthracenecarboxamide |
| A13 | 6248530 | N-(9,10-dioxo-9,10-dihydro-2-anthracenyl)-3,4,5-trimethoxybenzamide |
| A14 | 6722175 | N-9H-fluoren-2-yl-4-propoxybenzamide |
| A15 | 50675545 | N-[3-(1,2,3,4-tetrahydroisoquinolin-7-yl)phenyl]acetamide |
