# Supplementary material for: Substituted anthraquinones represent a potential scaffold for DNA methyltransferase 1-specific inhibitors
Source: PLoS One. 2019 Jul 15;14(7):e0219830. doi: 10.1371/journal.pone.0219830 (PMC6629088; doi:10.1371/journal.pone.0219830)
Supplement: S3 Table — 15 compounds (A1 –A15) were screened for inhibition of RFTS(-) DNMT1 at 10 μM and 50 μM. In all cases, reactions were conducted in triplicate. A matched reaction in the absence of DNMT1 was subtracted from each assay. The resulting corrected fluorescence data was averaged and fitted in Kaleidagraph to determine the initial velocity. Compounds were assayed in batches. Reported below are initial velocities for each condition (errors are from linear regression). To calculate percent activity, the initial velocity in the presence of inhibitor was divided by the initial velocity observed in the absence of inhibitor; errors from initial velocities were propagated. These percent activities are reported in Fig 1. (DOCX) [file pone.0219830.s006.docx]

**S3 Table. Initial velocity data from anthraquinone screen.** 15 compounds (A1 – A15) were screened for inhibition of RFTS(-) DNMT1 at 10 µM and 50 µM. In all cases, reactions were conducted in triplicate. A matched reaction in the absence of DNMT1 was subtracted from each assay. The resulting corrected fluorescence data was averaged and fitted in Kaleidagraph to determine the initial velocity. Compounds were assayed in batches. Reported below are initial velocities for each condition (errors are from linear regression). To calculate percent activity, the initial velocity in the presence of inhibitor was divided by the initial velocity observed in the absence of inhibitor; errors from initial velocities were propagated. These percent activities are reported in Fig 1.

|  | 10 µM Compound | 50 µM Compound |
| --- | --- | --- |
|  | Initial Velocity (RFU/min) | Initial Velocity (RFU/min) |
| DMSO | 96 ± 4 | 95 ± 3 |
| A1 | 86 ± 7 | 86 ± 4 |
| A2 | 95 ± 3 | 90 ± 6 |
| A3 | 51 ± 3 | 15 ± 12 |
| A4 | 92 ± 3 | 79 ± 5 |
| A5 | 87 ± 5 | 66 ± 4 |
| DMSO | 99 ± 5 | 98 ± 4 |
| A6 | 100 ± 7 | 95 ± 7 |
| A7 | 101 ± 3 | 89 ± 8 |
| A8 | 104 ± 8 | 69 ± 5 |
| A9 | 79 ± 5 | 47 ± 4 |
| A10 | 99 ± 5 | 44 ± 3 |
| DMSO | 101 ± 4 | 102 ± 6 |
| A11 | 104 ± 8 | 46 ± 2 |
| A12 | 99 ± 6 | 71 ± 4 |
| A13 | 102 ± 3 | 34 ± 8 |
| A14 | 86 ± 7 | 90 ± 3 |
| A15 | 104 ± 5 | 81 ± 4 |
