# Supplementary material for: Substituted anthraquinones represent a potential scaffold for DNA methyltransferase 1-specific inhibitors
Source: PLoS One. 2019 Jul 15;14(7):e0219830. doi: 10.1371/journal.pone.0219830 (PMC6629088; doi:10.1371/journal.pone.0219830)
Supplement: S4 Table — Compounds that inhibited fluorescence generation in the initial DNMT1 screen, were examined for their ability to inhibit the coupling enzyme used in the assay, Gla I. Reactions, conducted in triplicate, contained 50 μM compound. A matched reaction in the absence of Gla I was subtracted from each assay. The resulting corrected fluorescence data was averaged and fitted in Kaleidagraph to determine the initial velocity. Compounds were assayed in batches. Reported below are initial velocities for each condition (errors are from linear regression). To calculate percent activity, the initial velocity in the presence of inhibitor was divided by the initial velocity observed in the absence of inhibitor; errors from initial velocities were propagated. These percent activities are reported in Table 1. (DOCX) [file pone.0219830.s007.docx]

**S4 Table. Initial velocity data from Gla I counter screen.** Compounds that inhibited fluorescence generation in the initial DNMT1 screen, were examined for their ability to inhibit the coupling enzyme used in the assay, Gla I. Reactions, conducted in triplicate, contained 50 µM compound. A matched reaction in the absence of Gla I was subtracted from each assay. The resulting corrected fluorescence data was averaged and fitted in Kaleidagraph to determine the initial velocity. Compounds were assayed in batches. Reported below are initial velocities for each condition (errors are from linear regression). To calculate percent activity, the initial velocity in the presence of inhibitor was divided by the initial velocity observed in the absence of inhibitor; errors from initial velocities were propagated. These percent activities are reported in Table 1.

|  | Initial Velocity (RFU/min) |
| --- | --- |
| DMSO | 298 ± 13 |
| A2 | 290 ± 15 |
| A3 | 105 ± 11 |
| A5 | 203 ± 11 |
| A8 | 263 ± 26 |
| A9 | 155 ± 11 |
| DMSO | 292 ± 15 |
| A10 | 148 ± 14 |
| A11 | 283 ± 14 |
| A12 | 275 ± 21 |
| A13 | 277 ± 21 |
