# Supplementary material for: Substituted anthraquinones represent a potential scaffold for DNA methyltransferase 1-specific inhibitors
Source: PLoS One. 2019 Jul 15;14(7):e0219830. doi: 10.1371/journal.pone.0219830 (PMC6629088; doi:10.1371/journal.pone.0219830)
Supplement: S5 Table — Inhibition by compounds A11 and A13 was observed in the presence and absence of 0.01% Triton X-100 in the endonuclease-coupled DNA methylation assay. Triplicate corrected fluorescence data was averaged and fitted in Kaleidagraph to determine the initial velocity; error is from linear regression. Percent activity was determined by comparing to a DMSO-containing control assay. (DOCX) [file pone.0219830.s008.docx]

**S5 Table. Effect of Triton X-100 on observed inhibition of RFTS(-) DNMT1.** Inhibition by compounds A11 and A13 was observed in the presence and absence of 0.01% Triton X-100 in the endonuclease-coupled DNA methylation assay. Triplicate corrected fluorescence data was averaged and fitted in Kaleidagraph to determine the initial velocity; error is from linear regression. Percent activity was determined by comparing to a DMSO-containing control assay.

|  | Initial Velocity (RFU/min) | Percent Activity |
| --- | --- | --- |
| DMSO + Triton | 100 ± 4 | - |
| A11 | 66 ± 4 | 60 ± 4 |
| A11 + Triton | 69 ± 5 | 63 ± 5 |
| A13 | 59 ± 5 | 54 ± 5 |
| A13 + Triton | 62 ± 4 | 56 ± 3 |
