# Supplementary material for: Substituted anthraquinones represent a potential scaffold for DNA methyltransferase 1-specific inhibitors
Source: PLoS One. 2019 Jul 15;14(7):e0219830. doi: 10.1371/journal.pone.0219830 (PMC6629088; doi:10.1371/journal.pone.0219830)
Supplement: S6 Table — The concentration dependence of inhibition by compounds A11 and A13 was determined using the endonuclease-coupled DNA methylation assay. The concentration of inhibitor was varied from 0–100 μM. In all cases, reactions were conducted in triplicate. A matched reaction in the absence of RFTS(-) DNMT1 was subtracted from each assay. The resulting corrected fluorescence data was averaged and fitted in Kaleidagraph to determine the initial velocity. Percent activity was determined by comparing the initial velocity in the presence of inhibitor to the initial velocity observed in the absence of inhibitor. Percent activity from at least 3 independent experiments was averaged; standard deviation was used to calculate the error. Average percent activity data are plotted in Fig 3B. (DOCX) [file pone.0219830.s009.docx]

**S6 Table. IC_50_ determination.** The concentration dependence of inhibition by compounds A11 and A13 was determined using the endonuclease-coupled DNA methylation assay. The concentration of inhibitor was varied from 0 – 100 µM. In all cases, reactions were conducted in triplicate. A matched reaction in the absence of RFTS(-) DNMT1 was subtracted from each assay. The resulting corrected fluorescence data was averaged and fitted in Kaleidagraph to determine the initial velocity. Percent activity was determined by comparing the initial velocity in the presence of inhibitor to the initial velocity observed in the absence of inhibitor. Percent activity from at least 3 independent experiments was averaged; standard deviation was used to calculate the error. Average percent activity data are plotted in Fig 3B.

| [A11] (µM) |  |  |  | Average Percent Activity |
| --- | --- | --- | --- | --- |
| 4 | 94 | 108 | 99 | 100 ± 7 |
| 10 | 98 | 102 | 86 | 95 ± 8 |
| 25 | 82 | 68 | 76 | 75 ± 7 |
| 50 | 53 | 41 | 58 | 51 ± 9 |
| 100 | 36 | 23 | 29 | 29 ± 7 |

| [A13] (µM) |  |  |  |  | Average Percent Activity |
| --- | --- | --- | --- | --- | --- |
| 5 | 95 | 85 | 93 | 81 | 89 ± 7 |
| 15 | 69 | 70 | 60 |  | 66 ± 6 |
| 30 | 46 | 59 | 36 |  | 47 ± 12 |
| 50 | 33 | 29 | 36 | 31 | 32 ± 3 |
| 100 | 18 | 15 | 13 | 20 | 17 ± 3 |
