# Supplementary material for: Substituted anthraquinones represent a potential scaffold for DNA methyltransferase 1-specific inhibitors
Source: PLoS One. 2019 Jul 15;14(7):e0219830. doi: 10.1371/journal.pone.0219830 (PMC6629088; doi:10.1371/journal.pone.0219830)
Supplement: S7 Table — Inhibition of RFTS(-) DNMT1 by compounds A11 and A13 was assessed at 100 μM in the endonuclease-coupled DNA methylation assay. One set of assay solutions was immediately examined. Another set was allowed to incubate at room temperature for 60 minutes before addition of enzymes. In all cases, assays were conducted in triplicate and a matched reaction in the absence of DNMT1 was subtracted from each assay. Triplicate corrected fluorescence data was averaged and fitted in Kaleidagraph to determine the initial velocity. Percent activity was determined by comparing to the DMSO-containing control assay. (DOCX) [file pone.0219830.s010.docx]

**S7 Table. Examining the stability of A11 and A13.** Inhibition of RFTS(-) DNMT1 by compounds A11 and A13 was assessed at 100 µM in the endonuclease-coupled DNA methylation assay. One set of assay solutions was immediately examined. Another set was allowed to incubate at room temperature for 60 minutes before addition of enzymes. In all cases, assays were conducted in triplicate and a matched reaction in the absence of DNMT1 was subtracted from each assay. Triplicate corrected fluorescence data was averaged and fitted in Kaleidagraph to determine the initial velocity. Percent activity was determined by comparing to the DMSO-containing control assay.

|  | Assayed Immediately | | Incubated Before Assaying | |
| --- | --- | --- | --- | --- |
|  | Initial Velocity (RFU/min) | Percent Activity | Initial Velocity (RFU/min) | Percent Activity |
| DMSO | 98 ± 10 | - | 91 ± 5 | - |
| A13 | 15 ± 3 | 16 ± 4 | 13 ± 4 | 14 ± 4 |
| A11 | 28 ± 4 | 31 ± 5 | 30 ± 4 | 33 ± 5 |
