# Supplementary material for: Substituted anthraquinones represent a potential scaffold for DNA methyltransferase 1-specific inhibitors
Source: PLoS One. 2019 Jul 15;14(7):e0219830. doi: 10.1371/journal.pone.0219830 (PMC6629088; doi:10.1371/journal.pone.0219830)
Supplement: S8 Table — Fluorescence polarization was used to investigate the mechanism of inhibition by compounds A11 and A13. When the fluorophore-tagged DNA is bound by RFTS(-) DNMT1, the polarization value increases. Small molecules capable of competing with DNA for binding should decrease the observed polarization. Assays were conducted in triplicate. The average and standard deviation of the triplicate data is plotted in Fig 4B. (DOCX) [file pone.0219830.s011.docx]

**S8 Table. DNA-competitive binding using fluorescence polarization.** Fluorescence polarization was used to investigate the mechanism of inhibition by compounds A11 and A13. When the fluorophore-tagged DNA is bound by RFTS(-) DNMT1, the polarization value increases. Small molecules capable of competing with DNA for binding should decrease the observed polarization. Assays were conducted in triplicate. The average and standard deviation of the triplicate data is plotted in Fig 4B.

|  |  |  |  | Average Polarization (mP) |
| --- | --- | --- | --- | --- |
| DNA | 84 | 82 | 84 | 83 ± 1 |
| Complex | 200 | 206 | 212 | 206 ± 6 |
| +LCA | 88 | 88 | 84 | 77 ± 2 |
| +A13 | 152 | 158 | 154 | 155 ± 3 |
| +A11 | 121 | 119 | 118 | 119 ± 2 |
