# Supplementary material for: Substituted anthraquinones represent a potential scaffold for DNA methyltransferase 1-specific inhibitors
Source: PLoS One. 2019 Jul 15;14(7):e0219830. doi: 10.1371/journal.pone.0219830 (PMC6629088; doi:10.1371/journal.pone.0219830)
Supplement: S9 Table — Isozyme specificity was investigated using RFTS(+) DNMT1 and the C-terminal catalytic domain of DNMT3a. Activity of both enzymes was investigated using the endonuclease-coupled DNA methylation assay in the presence and absence of 100 μM inhibitor. In all cases, reactions were conducted in triplicate. A matched reaction in the absence of methyltransferase was subtracted from each assay. The resulting corrected fluorescence data was averaged and fitted in Kaleidagraph to determine the initial velocity. Compounds were assayed in batches. Reported below are initial velocities for each condition (errors are from linear regression). To calculate percent activity, the initial velocity in the presence of inhibitor was divided by the initial velocity observed in the absence of inhibitor; errors from initial velocities were propagated. These percent activities are reported in Table 3. (DOCX) [file pone.0219830.s012.docx]

**S9 Table. Isozyme specificity of A11 and A13 inhibition.** Isozyme specificity was investigated using RFTS(+) DNMT1 and the C-terminal catalytic domain of DNMT3a. Activity of both enzymes was investigated using the endonuclease-coupled DNA methylation assay in the presence and absence of 100 µM inhibitor. In all cases, reactions were conducted in triplicate. A matched reaction in the absence of methyltransferase was subtracted from each assay. The resulting corrected fluorescence data was averaged and fitted in Kaleidagraph to determine the initial velocity. Compounds were assayed in batches. Reported below are initial velocities for each condition (errors are from linear regression). To calculate percent activity, the initial velocity in the presence of inhibitor was divided by the initial velocity observed in the absence of inhibitor; errors from initial velocities were propagated. These percent activities are reported in Table 3.

|  | RFTS(+) DNMT1 | DNMT3a |
| --- | --- | --- |
|  | Initial Velocity (RFU/min) | Initial Velocity (RFU/min) |
| DMSO | 24.5 ± 0.4 | 26.6 ± 0.4 |
| A13 | 14.3 ± 0.5 | 27.3 ± 0.7 |
| A6 | 22.8 ± 0.8 | 25.4 ± 0.6 |
| DMSO | 24.3 ± 0.5 | 27.6 ± 0.4 |
| A11 | 15.8 ± 0.6 | 22.9 ± 0.4 |
| LCA | 5.2 ± 0.3 | 8.7 ± 0.3 |
